# Eco – epidemiology: challenges and opportunities for tomorrow's epidemiologists

**DOI:** 10.11604/pamj.2014.17.317.4080

**Published:** 2014-04-25

**Authors:** Luchuo Engelbert Bain, Paschal Kum Awah

**Affiliations:** 1 Centre for Population Studies and Health Promotion, Yaounde, Cameroon; 2 Department of Military Health, Ministry of Defence, Yaounde, Cameroon; 3 Faculty of Arts, Letters and Social Sciences, University of Yaounde I, Yaounde, Cameroon

**Keywords:** Eco–epidemiology, epidemiologists, challenges, opportunities

## Commentary

The study of the distribution and determinants of disease in populations and its application to the control of health problems is generally considered as epidemiology [1]. Sir Austin Bradford Hill's (1897-1991) contributions to epidemiology constitute a major breakthrough in the field of epidemiology. The risk factor paradigm from his perspective and inspired by his criteria of causation has made tremendous contributions to epidemiology [2]. The conceptual approach combining molecular, societal and population-based aspects to study a health-related problem is generally considered eco - epidemiology. John Snow and Joseph Goldberger's to an extent used the eco - epidemiological thought in analyzing public health concerns (Cholera and Pellagra) during an infectious disease era. They both recognized how social and biological changes could both interact to lead to disease. It is quite apparent that the identification of risk factors will not be sufficient for epidemiologists to confront some of the most pressing public health challenges of our time, such as those emanating from the HIV - AIDS pandemic, social inequalities, and movements of and changes in populations. It is often forgotten at times by academia however, that Hill himself realized the shortcomings of his criteria, insisting that these were mainly to guide the epidemiologist [2].

Advances in scientific thought and growing complexity of disease, the search of modern methods to better handle pressing public health concerns compels deep thought. One of the concepts that have been put forward with reasonable and promising gateways in addressing these problems in a holistic fashion could be the careful embracing by epidemiologists, an eco - epidemiological approach in subject (problem) analysis. Though it could demand more time, a larger team, and at times more money and might have to deal with lots of disagreements or mixed convictions, conclusions reached through this analytic approach could be almost definitive and save money and time for the future.

### Subject Analysis

The 13th World AIDS conference in South Africa in the year 2000 remains one of the major cornerstones as concerns the recognition and acceptance of the eco - epidemiological school of thought as an unavoidable tool in properly addressing the fight against HIV - AIDS. Recognition of social roles not only risk factors, women's rights and family relationships recognizing as key area to intervene in to reduce perpetuation of HIV, curb stigma and improve AIDS care. An overarching theme of the conference was to recognize causes on multiple levels and advance both the qualitative and quantitative methods required to investigate them. Throughout its history, epidemiology has undergone numerous methodological and conceptual developments, as well as changes in the scale at which epidemiologists focus their research [3]. Consequently, it has been characterized in terms of its eras, such as the “infectious disease era” and the “risk factor era” [4]. The place of interdisciplinary teams today in tackling and understanding health issues in a holistic fashion is compelling. The once neglected interconnections existing between the social, cultural, economic and physical environments in understanding the notion of disease, communicable and non - communicable is becoming more and more recognized in modern times epidemiology [3, 5]. Susser and Susser proposed breaking the constraints of the risk factor paradigm. They envisioned for the future an “eco-epidemiology” that would explicitly recognize multilevel causation and emphasize the ties that bind epidemiology to public health [6].

Reductionist approaches to research questions, focusing on proximate and linear cause-and-effect relationships, have characterized much of what epidemiology has contributed to public health in the second half of the 20th century, and a large array of important individual-level risk factors have been identified. However, this focus on individual risk factors has left understudied the forces that act at other levels to determine health [7], and has done little to prevent the recruitment of new high-risk individuals to replace those whose risks have been reduced. Also, only recently have eco-epidemiologists begun to grapple with the population health implications of the total ecological impress of human activity.

Nearly two decades later, Susser formally introduced levels of organization into epidemiology. He emphasized that the determinants of health on the individual level differ from those on the population level, despite the fact that populations comprise individuals, and that there are difficulties inherent to analytical movement between them [5].

### Where are we and where are we heading to?

The complexity of disease causation continues to get clearer and clearer every day. One concern put forth in these debates is that risk factor epidemiology-might be forcing epidemiology to focus more on individuals and less on populations and public health. Today, most epidemiologists acknowledge that public health is influenced by both population-level and individual-level determinants. Breakthroughs and rapid advancements in molecular biology, bacteriology, physiology, psychology and psychiatry with deeper understanding of impact of stress on the body systems is a great advantage and an opportunity to be seized by modern time epidemiologists to better apprehend, some once - upon - a - time complex diseases or health phenomena. Ecologic studies are valuable tools for generating hypotheses and addressing group-level determinants of disease risk. Traditional risk factor studies and genomic studies have helped establish the multifactorial concept of disease causation. Individual-level studies also have provided the biomedical community with hypotheses that have stimulated research into disease mechanisms that have led to reductions in morbidity and mortality for diseases such as HIV/AIDS, cardiovascular disease, and cancer [8].

Hence, the global context of population health comprises a variety of systems operating at various levels or scales, and each constitutes the environment for the other. As Soskolne et al state, we “must embrace greater complexity” because “the traditionally used, reductionist, linear approaches are inferior for understanding the interactive webs that are critical for sustainable development and for the health and well-being of future generations” [9]. With an escalating number of ecosystem changes taking effect across the planet, attention to developing and evaluating epidemiological methods that will generate data to inform policy that might help to change the present course is critical.

Recognizing these challenges, the term “eco-epidemiology” has been used to describe epidemiological research that embraces: the multi-level causation of disease, the importance of gene-environment interactions, the need to consider exposures over the life course and even over multiple generations, the utility of methodological cross-fertilization between communicable and chronic-disease-focused epidemiology, and the importance of broad contextual factors in the determination of population health states [3, 10].

Multilevel epidemiology calls for the study of health and disease determinants defined at the population level and individual level for a more comprehensive strategy to understanding human disease etiology. With the continued development of multilevel statistical methods and the advent of data mining, the technical constraints of the past will become less relevant to the next generation of epidemiologists who wish to embrace a more multilevel epidemiology [5, 6]. This paradigm encourages thinking about causes at multiple levels of organization and within the historical context of both societies and individuals. The proposed approach aims to preserve and build on the contributions of past eras, as well as the present one. Risk factor epidemiology is not undermined by eco - epidemiology but simply reinforces the idea of seeing it more holistically [11].

As our attention moves to multiple levels of the causal matrix of health determinants, there is an increasing interest in multilevel- and systems. Hence, a growing number of health experts argue that the health of a population can or must be viewed within the broader system of health determinants. It sounds logical and more realistic to situate populations not simply as a collection of individuals, but as being shaped by, and in turn shaping, the systematic context in which they operate [12]. Advances in genomics make it easier to see that the ecological perspective is relevant to understanding processes at the cellular and molecular level. Conceptual and methodological developments allow epidemiologists to study more thoroughly genetic and non-genetic causes alike. Consideration of the life course is increasingly well developed and defined as a key approach in eco - epidemiology (disease later in life could be due to early exposures - childhood, intrauterine etc).

Eco - epidemiology could predispose to too much specialization within the field of epidemiology (clinical, social, cultural, molecular, genetic epidemiology). Complexity in blending method and sound reason from these diverse schools of thought could be potentially problematic. However, the potential of eco - epidemiology to capture the desired spectrum of health determinants, that could explain both individual and population health concerns is almost conclusive. Susser gives equal weight to the hazards of the “*ecological fallacy*” (inferring causation at the individual level from population level comparisons) and the “*atomistic fallacy*” (inferring causation at the population level from individual level comparisons) [5].

The task before us is to draw together these different domains and then extract the implications for public health. An integrated approach to investigating disease and its prevention will necessarily subsume levels of causation, life course trajectories, kinds of causes, and types of diseases [3, 5, 13].

In conclusion: Despite the almost conclusive potential of an eco - epidemiological thought as a more suited and effective way to practice epidemiology in a modern world, the risk factor paradigm is the dominant if not exclusive focus of training in epidemiology. Multidisciplinary teams of epidemiologists, geneticists, molecular biologists and social scientists in appreciating public health concerns could be relatively very expensive and tedious to establish and coordinate. Though potentially beneficial from both economic and scientific standpoints in the long run, eco - epidemiology as new science must not in any way put the cause - effect paradigm in active search of causality, as proposed by Hill “in the shadows”. It would be most beneficial to public health if academia could start inculcating the “eco” in appreciation of health problems as multidisciplinary to future epidemiologists. There exists however a risk of lessening a deeper sense of sound reasoning that could arise from over reliance on other colleagues from allied disciplines.
